# Systematically programmed adaptive evolution reveals potential role of carbon and nitrogen pathways during lipid accumulation in *Chlamydomonas reinhardtii*

**DOI:** 10.1186/s13068-014-0117-7

**Published:** 2014-09-06

**Authors:** Natarajan Velmurugan, Minji Sung, Sung Sun Yim, Min S Park, Ji Won Yang, Ki Jun Jeong

**Affiliations:** Department of Chemical and Biomolecular Engineering (BK21+ Program), KAIST, 291 Daehak-ro, Yuseong-gu Daejeon, 305-701 Republic of Korea; Bioscience Division, Los Alamos National Laboratory, Bikini Atoll Road, Los Alamos, NM 87545 USA; KI for the Biocentury, KAIST, 291 Daehak-ro, Yuseong-gu Daejeon, 305-701 Republic of Korea

**Keywords:** *Chlamydomonas reinhardtii*, Adaptive evolution, Flow cytometry, Proteomics

## Abstract

**Background:**

The concept of adaptive evolution implies underlying genetic mutations conferring a selective advantage to an organism under particular environmental conditions. Thus, a flow cytometry-based strategy was used to study the adaptive evolution in *Chlamydomonas reinhardtii* wild-type strain CC124 and starchless mutant sta6-1 cells, with respect to lipid metabolism under nitrogen-(N) depleted and -replete conditions.

**Results:**

The successive sorting and regeneration of the top 25,000 high-lipid content cells of CC124 and sta6-1, combined with nitrogen starvation, led to the generation of a new population with an improved lipid content when compared to the original populations (approximately 175% and 50% lipid increase in sta6-1 and CC124, respectively). During the adaptive evolution period, the major fatty acid components observed in cells were C16:0, C16:1, C18:0, and C18:1-3, and elemental analysis revealed that cellular carbon to nitrogen ratio increased at the end of adaptive evolution period In order to gain an insight into highly stimulated intracellular lipid accumulation in CC124 and sta6-1 resulting from the adaptive evolution, proteomics analyses of newly generated artificial high-lipid content populations were performed. Functional classifications showed the heightened regulation of the major chlorophyll enzymes, and the enzymes involved in carbon fixation and uptake, including chlorophyll-ab-binding proteins and Rubisco activase. The key control protein (periplasmic L-amino acid oxidase (LAO1)) of carbon-nitrogen integration was specifically overexpressed. Glutathione-S-transferases and esterase, the enzymes involved in lipid-metabolism and lipid-body associated proteins, were also induced during adaptive evolution.

**Conclusions:**

Adaptive evolution results demonstrate the potential role of photosynthesis in terms of carbon partitioning, flux, and fixation and carbon-nitrogen metabolism during lipid accumulation in microalgae. This strategy can be used as a new tool to develop *C. reinhardtii* strains and other microalgal strains with desired phenotypes such as high lipid accumulation.

**Electronic supplementary material:**

The online version of this article (doi:10.1186/s13068-014-0117-7) contains supplementary material, which is available to authorized users.

## Background

*Chlamydomonas reinhardtii*, a unicellular green alga, has been investigated intensely as a robust model system for the formation of intracellular lipid bodies that contain triacylglycerol (TAG) [1-3]. The intracellular lipid body formation in *C. reinhardtii* depends on several factors including stress conditions such as nutrient starvation, temperature, salinity, and light intensity [4]. In the past few years, researchers have investigated the intracellular lipid accumulation in microalgae under different stress conditions [1,5]. However, the molecular mechanisms of lipid accumulation in relation with carbon and nitrogen metabolisms, and cell division remain poorly understood in microalgae. Detailed studies on the molecular mechanism of lipid accumulation in microalgae under stress conditions should facilitate improvements in the lipid productivity, cultivation processes, and strain development for biofuels production [4]. The cellular physiology of *C. reinhardtii* changes depending on nitrogen availability [6]. Under nitrogen-depleted conditions, neutral lipids and starch will accumulate to high levels to serve as a primary form of energy storage [4].

Among several *C. reinhardtii* strains, CC124 wild-type and sta6-1 mutants are broadly studied. The starchless sta6-1 mutants of *C. reinhardtii* have been reported to produce a higher level of TAG than the wild-type under nitrogen-depleted conditions [5]. The sta6-1 mutants are deficient of a central starch synthesis enzyme (ADP-glucose pyrophosphorylase) and accumulate less than 1% of the starch compared with the wild-type under nitrogen-depleted conditions [5]. This deficiency impacts carbon metabolism and flow which may induce accumulation of noticeable levels of TAG in sta6-1 mutants [6]. It has been proven that adaptive mutation plays an important role in the evolution of microorganisms [7]. Adaptive mutations have been reported in some microbes, such as bacteria and yeast, but not well-known for microalgae [8]. It has been reported that the evolution of the microalgae *Dictyosphaerium chlorelloides* has caused it to adapt to an environment containing the highly toxic material 2,4,6,-trinitrotoluene (TNT) [7]. We therefore studied the mechanism of adaptation by *C. reinhardtii* wild-type and sta6-1 mutant strains under nitrogen-depleted and -replete conditions using flow cytometry. A study by Ramanan *et al*. [9] proposed a separate chloroplast pathway for TAG synthesis in starchless mutants of *C. reinhardtii* strains. We performed adaptive evolution of wild-type CC124 and sta6-1 mutants from a TAG accumulation perspective which included nitrogen starvation, time course, cell density-dependent adaptive evolution, and the overall yields of lipids, elemental composition, and proteomic analyses.

Flow cytometry enables the analysis of the different features or physiological states of microalgae at the single-cell level. Cells with a specific characteristic can be separated from the heterologous population for growth or analysis using fluorescence-activated cell sorting (FACS) [10]. Enhanced production of lipid bodies can be achieved by optimization of the production processes of microalgae, or the selection of strains with improved features or overproducers [11]. The unique ability of microalgae to adapt their metabolism to various culture conditions provides opportunities to modify and maximize the lipid production [11]. For example adaptive responses, which help microalgae to survive under environmental stresses, can cause the algae to maximize the lipid content including polyunsaturated fatty acids [12]. Therefore, gaining more detailed information on the underlying regulatory mechanisms is necessary to devise and implement such production strategies [13]. More recently we have examined and compared intracellular lipid body accumulation in *C. reinhardtii* wild-type and mutant sta6-1 strains by flow cytometry [14]. Using different fluorescent dyes in combination with FACS, we can isolate microalgal strains that produce high levels of lipid in certain stress conditions or after treating them with different mutagens. Doan and Obbard [15], and Montero *et al*. [16] isolated lipid-overproducing *Nannochloropsis* and *T. suecica* strains, respectively, using lipophilic fluorescent dye Nile Red in combination with FACS. Recently, we have demonstrated that the fluorescent dye BODIPY 505/515, in combination with FACS, allows single-cell level isolation and regeneration of algal strains that possess a high lipid content [14].

The recent genome sequencing of *C. reinhardtii* helps us to understand the molecular mechanisms of lipid body formation, TAG synthesis, and accumulation in microalgae [2,6]. Comprehensive proteomics analysis provides clear insight into the mechanisms of microalgal lipid biosynthesis. Proteomics analyses have been reported for chloroplast, mitochondria, and lipid bodies [17-20]. However, proteomics analysis of the total proteome of *C. reinhardtii* has so far been lacking. In this study, our primary focus is developing a flow cytometry-based strategy to address adaptive evolution and strains development with respect to high lipid accumulation. To this effect, we established a unique flow cytometry-based adaptive evolution procedure for *C. reinhardtii* wild-type and mutant sta6-1 strains that have been subjected to nitrogen limitation conditions for over 50 days. Specifically, we analyzed the total proteomics of *C. reinhardtii* strains to determine their metabolic response to the adaptive evolution.

## Results and discussion

### Physiological behavior of wild-type and mutant sta6-1 during adaptive evolution under nitrogen-depleted and -replete conditions

**Figure 1:**
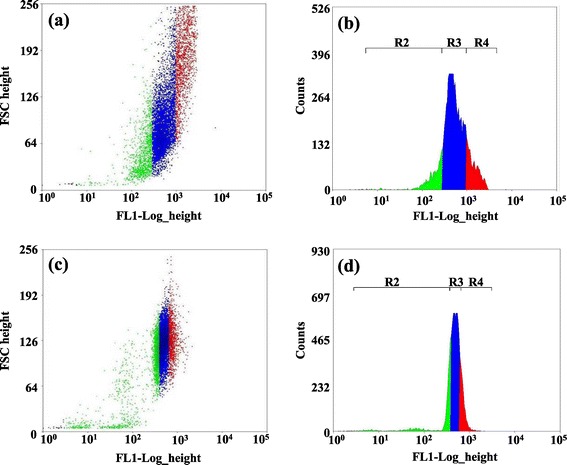
**Two dimensional dot plots (a, c) and flow cytograms (b, d) of BODIPY 505/515-stained 0 hour seed cells of CC124 (a, b) and sta6-1 (c, d).** Region R2 (green), R3 (blue), and R4 (red) in flow cytograms represent low, medium, and high lipid content cells, respectively. A total of 25,000 cells from the top 2 to 3% of R4 region were sorted and regenerated in nitrogen depletion and repletion TAP medium, separately. The mean values of the histograms of the seed cells of CC124 and sta6-1 were 662.11 and 494.35, respectively. Abbreviations: BODIPY, 4,4-difluoro-1,3,5,7-tetramethyl-r-bora-3a,41-diaza-s-indacene.

The primary objective of this study was to specifically select and regenerate the high-lipid content cells of *C. reinhardtii* strains from a heterogeneous population of cells that contain a wide range of lipid contents (Figure 1). Microalgal cellular growth and metabolic pathways respond in a highly dynamic way to environmental conditions, especially nutrient availability [21]. To obtain a healthy population (red-color region in Figure 1), the seed cultures of wild-type CC124 and mutant sta6-1 were obtained from the late-log phase in nitrogen-supplemented Tris Acetate Phosphate liquid Medium (TAP) medium. The initial seed cultures for inoculum were maintained around approximately 2.0 × 10^6^ cells ml^1^ in TAP medium. The lipophilic fluorescent dye BODIPY 505/515 was used to stain the wild-type CC124 and mutant sta6-1 for flow cytometry screening and cell sorting as described previously [14]. Prior to sorting (0 hours), samples were taken immediately after re-suspension in TAP medium. Since our aim is to isolate lipid overproducing strains, 25,000 cells from the high-lipid content population of BODIPY 505/515-stained seed cells of CC124 and sta6-1 were sorted directly into TAP medium and were re-suspended in nitrogen-depleted or -replete TAP medium separately (in triplicate).

**Figure 2:**
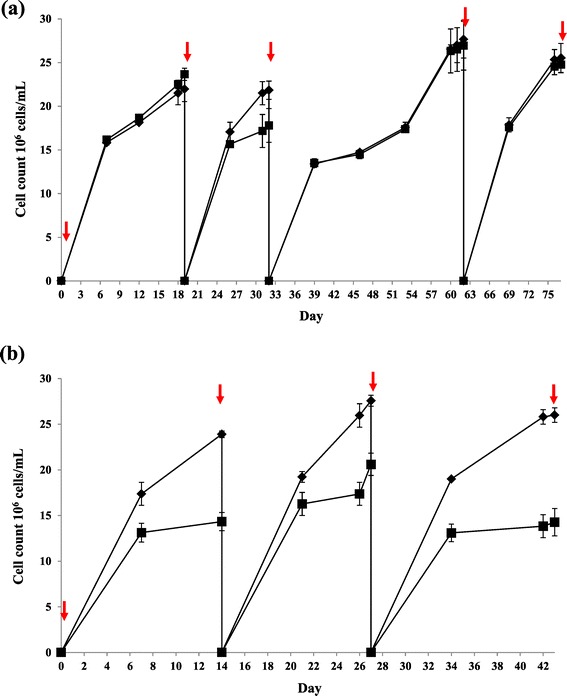
**Growth curve (cell count) for CC124 (a) and the sta6-1 (b) strains, in nitrogen-depletion (diamond) and -repletion (square) TAP medium.** Each data point represents three replicates. Arrows indicate the inoculation points of the 25,000 cells from the top 2% of cells sorted using flow cytometry (Day 0 indicates inoculation of 25,000 cells from the top 2% of 0 hour seed culture). Abbreviations: TAP, Tris Acetate Phosphate liquid Medium.

At the beginning of evolution period, the wild-type CC124 was found to reach high cell density (or late-stationary phase) by day 19 (Figure 2). The mutant sta6-1 showed high cell density (or late-stationary phase) by day 14 (Figure 2). Cells from day 19 and day 14 cultures of strains CC124 and sta6-1 respectively were considered as first generation cultures and prepared for next consecutive rounds of cell sorting. A total of 25,000 cells from the top 2% were sorted using flow cytometry. Sorted cells from each round (at indicated intervals) were used for inoculation of a new culture and the remaining portions of the cells were used for the measurement of total lipid content, microscopic analysis, elemental analysis, and proteomics analysis. After three rounds of sorting, the wild-type CC124 was shown to have relatively slow growth compared with earlier sorting periods, and finally reached high cell density by day 62. A possible reason for the slow growth at this time point (from day 32 to day 62) is that during the sorting, cells may partially affect by the fluid acceleration, electrical or mechanical shock, and optical stress and that caused the slower growth of sorted cells at the initial stage. However, the cells showed healthy growth after the initial stage and reached high cell density by day 62. In our adaptive evolution experiments, we kept the 0 hour seed and first generation cultures as positive controls. The lipid accumulation and microalgal growth performance of further generations were compared with 0 hour (seed cultures) and first generation positive controls. The cell concentrations of CC124 and sta6-1 in the presence and absence of nitrogen were measured and shown in Figure 2. The starchless strain sta6-1 had attained the highest cell concentrations in nitrogen-depleted condition rather than nitrogen-replete conditions during the adaptive evolution period. This indicates that the rate of arrest of cell division in nitrogen-depleted conditions was relatively lower than nitrogen-replete conditions during the adaptive evolution period. The average highest cell concentration for both strains were found to be approximately 25 × 10^6^ cells ml^−1^ in nitrogen-depleted condition during the adaptive evolution period (Figure 2). The wild-type CC124 showed overall similar cell concentrations in nitrogen-depleted and -replete conditions at all indicated times (Figure 2a). This indicates that wild-type CC124 cell divisions and regenerations are similar in both media regardless of whether nitrogen is present or not. However, the starchless mutant sta6-1 showed a different growth pattern, as relatively fewer cell concentrations were observed in nitrogen-replete conditions at all indicated times (Figure 2b). It is interesting to observe that the mutant strains have superior growth in nitrogen-depleted conditions than nitrogen-replete conditions.

### Lipid accumulation in *C. reinhardtii* strains during adaptive evolution

**Figure 3:**
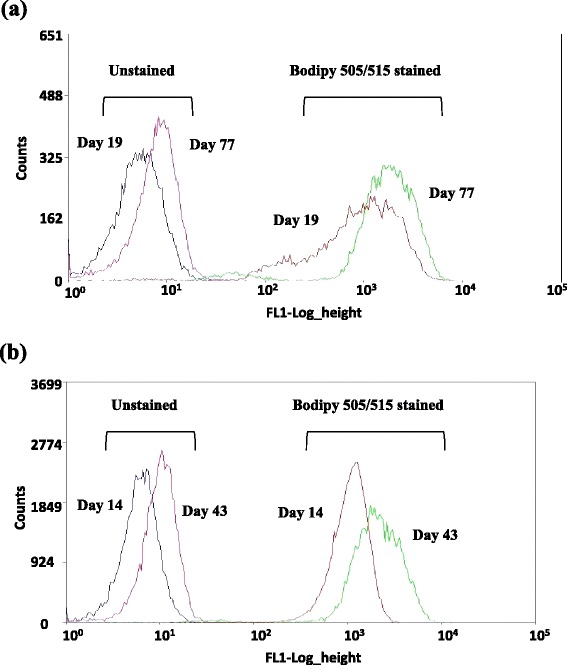
**Flow cytograms of adaptive evolved strains of CC124 (a) and sta6-1 (b) in nitrogen-depletion TAP medium. (a)** CC124 - Blue and purple representing unstained cells of day 19 and 77, respectively. Red and green representing BODIPY 505/515-stained cells of day 19 and 77, respectively. **(b)** sta6-1 - Blue and purple representing unstained cells of day 14 and 43, respectively. Red and green representing BODIPY 505/515-stained cells of day 14 and 43, respectively. The mean values of histograms of unstained and BODIPY 505/515-stained cells of CC124 of day 19 and 77 were 6.7, 10.98, 1724.45, and 2206.01, respectively. The mean values of histograms of unstained and BODIPY 505/515-stained cells of sta6-1 of day 14 and 43 were 5.2, 9.54, 1138.34, and 2427.78, respectively. Abbreviations: TAP, Tris Acetate Phosphate liquid Medium; BODIPY, 4,4-difluoro-1,3,5,7-tetramethyl-r-bora-3a,41-diaza-s-indacene.

*C. reinhardtii* CC124 and mutant sta6-1 were periodically collected during the course of adaptive evolution at indicated times and were stained with BODIPY 505/515 for FACS analysis of intracellular lipid bodies. The lipophilic dye BODIPY 505/515 can be used as a vital stain for the detection of neutral lipid bodies in the *C. reinhardtii* strains. Recently, BODIPY-flow cytometry-based protocol has been used to evaluate the lipid accumulation performance between wild-type and transgenic mutants of the diatom *Thalassiosira pseudonana* [22]. Figure 3 shows the autofluorescence intensities for unstained cells, along with the FL1 signals of BODIPY 505/515-stained CC124 and sta6-1. Flow cytograms from each sorting round for CC124 and sta6-1 are representative of the green fluorescence (FL1) from the first to last rounds of sorting for CC124 and sta6-1 during the adaptive evolution period in nitrogen-depleted conditions. We found that wild-type CC124 required a longer time to attain highest lipid accumulation than the starchless mutant sta6-1. The mean fluorescence intensity (FL1) from BODIPY staining of CC124 peaked on day 77 and similar autofluorescence intensity results were observed in nitrogen-depleted conditions (Figure 3a). The mutant sta6-1 attained the highest lipid content more rapidly compared to CC124, as it only took 43 days to reach peak autofluorescence intensity, which was observed throughout nitrogen-depleted conditions (Figure 3b). However, after further sorting and regeneration, CC124 and sta6-1 strains were found to not be accumulating more lipids (data not shown). This could be due to the fact that the maximum level of lipid accumulation had been achieved at or near those days during adaptive evolution, and spontaneous mutations might have caused cells to return to their original phenotype under some selective conditions [8]. Figure 3 presents a sequence of autofluorescence and fluorescence histograms related to the increased lipid accumulation in CC124 and sta6-1 during adaptive evolution. It is evident that there was a well-defined shift of autofluorescence as well as BODIPY 505/515-stained cells from the initial day to the final day of adaptation. This indicates the progressive movement of selected microalgal populations towards a significantly higher level of lipid accumulation in nitrogen starvation conditions during the adaptive evolution period. However, wild-type CC124 and mutant sta6-1 were unable to achieve progressive lipid accumulation in nitrogen-replete conditions (FACS data not shown, please refer to the lipid content measurement data in nitrogen-replete conditions in Table 1).

**Table 1:** **GC quantification of FAME content of adaptive evolved strains of CC124 and sta6-1 at different stages**

**Strain**	**Different stages (Day)**	**Nitrogen-repletion conditions**	**Nitrogen-depletion conditions**	**Fatty acid (% of total fatty acids in nitrogen-depletion conditions)** ^**a**^								
		**Total lipid (%)**	**Total lipid (%)**	**C14:0**	**C14:1**	**C15:0**	**C15:1**	**C16:0**	**C16:1**	**C17:1**	**C18:0**	**C18:1-3 (n)** ^**b**^
CC124	19	6.6	10.5	0.3	0.2	0.2	0.3	39.3	2.7	2.7	5.4	45.3
	32	5.9	13.5	0.3	0.0	0.2	0.2	40.1	4.5	2.8	3.4	45.3
	62	5.3	11.4	0.2	0.0	0.2	0.4	51.7	0.7	2.0	8.5	34.8
	77	7.0	15.9	0.2	0.0	0.2	0.4	40.7	0.5	1.2	8.9	46.4
sta6-1	14	5.8	5.9	0.4	2.0	0.6	0.8	85.3	2.7	0.7	5.2	45.0
	27	6.7	8.1	1.1	0.0	0.5	0.7	40.6	1.2	1.8	5.0	47.0
	43	7.4	14.0	0.2	0.0	0.1	0.3	48.9	0.6	2.4	11.3	35.8

^a^All data are expressed as mean quantity of triplicate experiments.

^b^C18:1n9c, C18:2n6t, C18:3n6c. Abbreviations: GC, Gas chromatography; FAME, Fatty acid methyl esters.

To confirm the conclusions drawn from the FACS analysis, the fatty acid methyl esters (FAME) contents of adaptive evolved CC124 and sta6-1 strains were quantified at all indicated time points by gas chromatography. Table 1 shows the separation by GC of FAME, prepared from total lipids of *C. reinhardtii* strains, comparison of the fatty acid composition of the TAG fraction, and total lipids. During the adaptive evolution period, the major fatty acid components observed in CC124 and sta6-1 cells were C16:0, C16:1, C18:0, and C18:1-3 (Table 1). Sequential increase of lipid content in the adaptive evolution period indicates the intergenerational stability of lipid levels of sorted CC124 and sta6-1 strains. The presence of palmitic, palmitoleic, stearic, and linoleic acids indicated a stable fatty acid composition (Table 1). C18:0 and C18:1-3 profiles of adaptive evolution strains of CC124 and sta6-1 showed a gradual increase throughout the adaptation. After adaptive evolution, CC124 and sta6-1 showed a 50% and 175% increase (10.52 to 15.9% and 5.94 to 14.04%) of lipids, respectively. As shown in FACS analysis (Figure 3), the mutant sta6-1 achieved the highest lipid (15.9%) content within 43 days. On the other hand, under nitrogen-replete conditions, the mutant sta6-1 showed relatively little increase in lipid content (5.84 to 7.42%), and wild-type CC124 showed no increase in lipid content.

**Figure 4:**
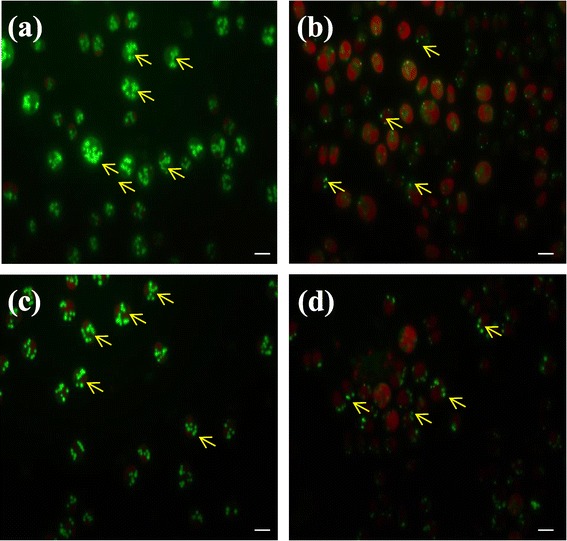
**Confocal images of BODIPY 505/515-stained adaptive evolved** ***Chlamydomonas reinhardtii*** **strains. (a)** CC124 in nitrogen-depleted conditions at day 77; **(b)** CC124 in nitrogen-replete conditions at day 77; **(c)** sta6-1 in nitrogen-depleted conditions at day 43; **(d)** sta6-1 in nitrogen-replete conditions at day 43. Arrows indicate lipid droplets. Each scale bar indicates 5 μm.

The lipid accumulation in the cells that went through adaptive evolution was analyzed with confocal microscope. Figure 4 shows confocal images of BODIPY 505/515-stained cells of CC124 and sta6-1 after adaptive evolution. The lipophilic property of the dye facilitates the BODIPY 505/515 accumulation in intracellular lipid compartments of the cell [23]. The strong red autofluorescence allowed the chlorophyll content to be easily distinguished from the lipid content (Figure 4). The confocal microscopy showed that the numbers of intracellular lipid bodies massively increased at the end of adaptive evolution (more than 22 intracellular lipid bodies were found per cell in adapted CC124 and sta6-1) in nitrogen-depleted condition (Figure 4a and c). Recently, Goodson *et al*. [1] reported that sta6 mutants had a range of 6 to 25 lipid bodies per cell in nitrogen starvation conditions, with an acetate boost. The large increase in numbers of intracellular lipid bodies in the adaptively evolved CC124 and sta6-1 strains under nitrogen-depleted conditions is a new observation. Meanwhile, the numbers of intracellular lipid bodies did not increase in CC124 and sta6-1 under nitrogen-replete condition (Figure 4b and d).

As shown in Figure 4b and 4d, the chlorophyll content in the adapted cells of CC124 and sta6-1 was found to be at a steady level during cultivation under nitrogen-replete conditions. These observations are in agreement with recent findings regarding the chlorophyll content in CC124 and sta6 in a nitrogen-replete medium [5]. Meanwhile, the chlorophyll content was significantly reduced in cells of CC124 and sta6-1 in nitrogen-depleted medium at the end of the adaptive evolution period (Table 2). Several researchers have also reported that the chlorophyll content of *C. reinhardtii* decreased under nitrogen limitation conditions [5,21,24,25]. It is probable that the accelerated loss of chlorophyll in the starchless mutants of *C. reinhardtii* is associated with the more severely attenuated O_2_ evolution activities observed in these strains [5]. Due to reduced levels of chlorophyll, less light energy is absorbed to be used for carbon fixation and cell division [21].

**Table 2:** **Autofluorescence of chloroplast of adaptive evolved strains CC124 and sta6-1 at different stages**

**Strain**	**Different stages (Day)**	**Mean fluorescence intensity (FL5 channel) (arbitrayunits)** ^**a**^	
		**Unstained**	**BODIPY 505/515 stained**
CC124	19	24.8	22.2
	77	17.0	16.4
sta6-1	14	15.9	16.3
	43	11.7	11.4

^a^All data are expressed as mean quantity of triplicate experiments. Abbreviations: BODIPY, 4,4-difluoro-1,3,5,7-tetramethyl-r-bora-3a,41-diaza-s-indacene.

Elemental analysis revealed that the cellular carbon to nitrogen ratio (C:N) increased at the end of adaptive evolution period (from day 19 to day 77 and day 14 to day 43, in CC124 and sta6-1, respectively) (Table 3). At day 19 and day 77 for CC124 strain, the C:N ratio was 9.5:1 and 11.1:1, respectively. At day 14 and day 43 for sta6-1 strain, the C:N ratio was 10.3:1 and 11.8:1, respectively (Table 3). This increase in C:N could also be explained in the context of an increase in lipid accumulation [26]. A unicellular marine diatom *Phaeodactylum tricornutum* reported an increase in C:N ratio as the intracellular lipid accumulation increased [26]. However this C:N ratio did not influence specific intracellular cellular carbohydrates [26].

**Table 3:** **Elemental analysis of adaptive evolved strains CC124 and sta6-1 at different stages**

**Strain**	**Different stages (Day)**	**Biochemical elements** ^**a**^				**C:N ratio**
		**C**	**N**	**H**	**S**	
CC124	19	47.8 ± 0.14	5.0 ± 0.03	7.1 ± 0.1	0.3 ± 0.01	9.5:1
	32	49.2 ± 0.1	5.3 ± 0.01	7.2 ± 0.004	0.4 ± 0.001	9.2:1
	62	50.3 ± 0.13	4.6 ± 0.02	7.5 ± 0.02	0.3 ± 0.002	10.9:1
	77	51.0 ± 0.13	4.6 ± 0.04	7.5 ± 0.02	0.3 ± 0.003	11.1:1
sta6-1	14	49.2 ± 0.04	4.8 ± 0.01	7.2 ± 0.03	0.3 ± 0.01	10.2:1
	27	47.2 ± 0.1	5.9 ± 0.0004	6.9 ± 0.002	0.3 ± 0.01	8.0:1
	43	52.5 ± 0.2	4.5 ± 0.02	7.8 ± 0.03	0.3 ± 0.01	11.8:1

^a^All data are expressed as mean quantity of triplicate experiments. Abbreviations: C:N, Carbon:Nitrogen ratio; C, Carbon; N, Nitrogen, H, Hydrogen; S, Sulfur.

### Molecular response of *C. reinhardtii* for adaptive evolution

To understand the molecular mechanisms of adaptive evolution under nitrogen starvation, we performed comprehensive proteomics analyses of the adaptively evolved cells of CC124 and sta6-1. At all indicated time points, lyophilized cells (nitrogen-depleted conditions) of adaptive evolved CC124 and sta6-1 were used for total protein extraction. The extracted protein materials were separated by two-dimensional gel electrophoresis using two different pH ranges (pH 3 to 10 and 4 to 7), separately. All scanned two-dimensional gel images of proteomes are provided in supplementary information (Additional file 1: Figure S1, Additional file 2: Figure S2, Additional file 3: Figure S3, and Additional file 4: Figure S4). Interestingly, we observed that the numbers of protein spots expressed between pH range 4 to 7 gradually increased in line with the adaptive evolution period for CC124 and sta6-1 (Table 4, Additional file 3: Figure S3, and Additional file 4: Figure S4). A recent report by Choi *et al*. [27] studied the comparative proteomic response of lipid metabolism in *C. reinhardtii* between pH range 4 to 10 using lipid over- and underproducing strains. In addition, the majority of earlier reports have been heavily centered at various specific targets (proteomic characterization of mitochondria [17], chloroplast [18], and lipid bodies [19]). While preparing this paper, Wase *et al*. [28] reported the protein profiling of nitrogen stress response in the *C. reinhardtii* strain using iTRAQ (isobaric Tags for Relative and Absolute Quantitation) methodology. However, the protein profiling of *C. reinhardtii* strains had not been characterized between different pH ranges. Thus, the observation of high amounts of accumulation of protein spots between acidic to neutral ranges (pH 4 to 7) is a new observation for *C. reinhardtii* CC124 and mutant sta6-1.

**Table 4:** **List of numbers of protein spots identified between pH range 4 to 7 for adaptive evolved cells of CC124 and sta6-1**

**Strain**	**Different stages (Day)**	**Number of protein spots**
CC124	19	578
	32	785
	62	805
	77	876
sta6-1	14	598
	27	759
	43	969

**Figure 5:**
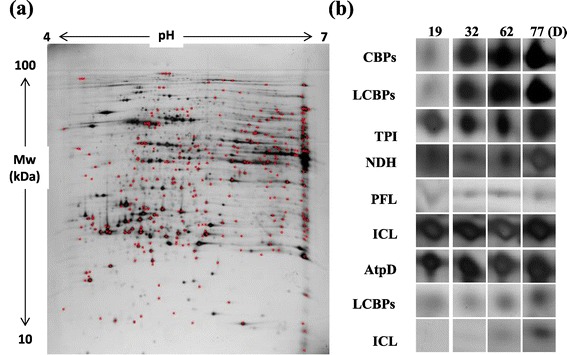
**Proteome profiles of CC124 during adaptive evolution period. (a)** The proteins showing differentially expressed levels during the adaptive evolution period are indicated as red points. **(b)** Of these, nine zoomed in spot areas highlighted from the day 19 profile gel are compared to corresponding protein spots of the day 32, 62, and 77 profile gels (comparison of all 20 proteins spots is provided in Additional file 5: Figure S5). The abbreviations for the enzymes included are as follows: CBP, chlorophyll-ab-binding proteins; LCBP, light harvesting proteins; TPI, triose phosphate isomerase; NDH, NADP depended-malate dehydrogenase; PFL, pyruvate-formate lyase; ICL, isocitrate lyase; AtpD, ATP synthase CF1 beta subunit.

**Figure 6:**
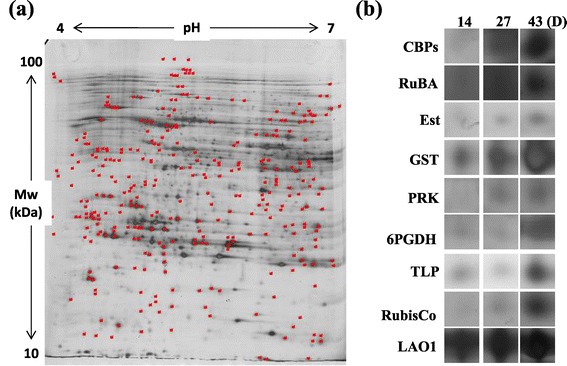
**Proteomic profiles of sta6-1 during adaptive evolution period. (a)** The proteins showing differentially expressed levels during the adaptive evolution period are indicated as red points. **(b)** Of these, nine zoomed in spot areas highlighted from the day 14 profile gel are compared to corresponding protein spots of the day 27 and 43 profile gels (comparison of all 24 proteins spots are provided in Additional file 6: Figure S6). The abbreviations for the enzymes included are as follows: CBP, chlorophyll-ab-binding proteins; RuBA, rubisco activase; Est, esterase; GST, glutathione-S-transferases; PRK, Phosphoribulokinase Rubisco activase; 6PGDH, 6-phosphogluconate dehydrogenase; TLP, thylakoid lumen protein; RubisCo, Ribulose-1,5-biphosphate carboxylase/oxygenase large subunit; LAO1, periplasmic L-amino acid oxidase catalytic subunit.

An extensive two-dimensional gel image analysis was carried out using PDQuest (Version 7.0, Bio-Rad, Hercules, CA, USA) software and differentially expressed protein profiles were identified at different time points during the adaptive evolution. After an extensive examination of image analysis results, proteins which exhibited a fold change difference of greater or less than 3-fold, at different time points of adaptive evolution, were considered to be significantly up- or down-regulated. We selected 60 significantly up- or down-regulated spots (common spots for both wild-type CC124 and mutant sta6-1) for protein identification using MALDI-TOF (Matrix-Assisted Laser Desorption-Ionization Time-of-Flight), and out of these we were able to identify 44 protein spots (Figures 5 and 6). The spots were selected based on their gradual differential expression patterns and specific differences in response to the adaptive evolution period; therefore it is conceivable that the differential expression was due to adaptive evolution. Among 44 spots, glutathione S-transferase (GST), Rubisco activase (RuBA), isocitrate lyase (ICL), mitochondrial translation factor Tu (mtTF-Tu), periplasmic L-amino acid oxidase catalytic subunit (LAO1), and mitochondrial carbonic anhydrase β-type (mtCA) were found twice. Among 44 spots, four proteins including protein phosphates (PPI), Ran-like small GTPase (GTPase), mitochondrial translation factor (mtTF-Tu), and mitochondrial carbonic anhydrase (mtCA) were found to be down-regulated, but all the other proteins including esterase (EST), pyruvate-formate lyase (PFL), and Rubisco exhibited up-regulation during adaptation (Figures 5 and 6). All spot images of the identified proteins during the adaptations are provided in Additional file 5: Figure S5 and Additional file 6: Figure S6.

**Figure 7:**
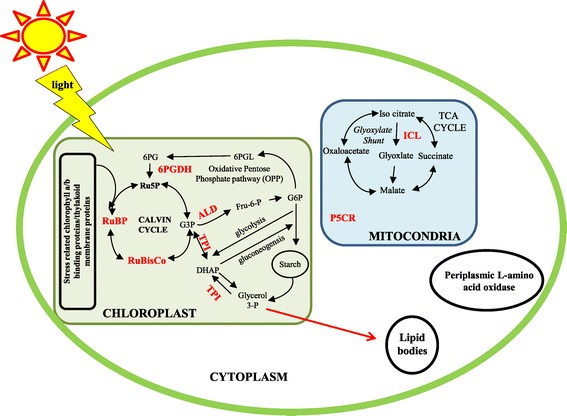
**Schematic diagram of up-regulated genes in their respective pathways in** ***Chlamydomonas reinhardtii*** **strains during adaptive evolution.** The up-regulated proteins in the proteomic study are indicated in red and bold.

Functional classification showed that the differentially expressed proteins were involved in photosynthesis, mitochondrial metabolism, lipid metabolism, vesicle trafficking, and other functions (Additional file 7: Table S1). Most of the identified proteins were chloroplast, mitochondrial, and lipid-related proteins [19,20]. Observations of an increased number of differentially expressed chloroplast proteins (especially higher abundance and up-regulation of Calvin cycle enzymes) revealed the potential role of carbon uptake and fixation pathways during lipid accumulation in *C. reinhardtii* strains (Figure 7). It is clear that stress conditions significantly modulate photosynthesis and anabolic processes in the *C. reinhardtii* strains [5]. Under some circumstances, the intracellular mechanisms are activated to sense total nitrogen levels in the medium in order to balance the use of nitrogen for protein or nucleotide biosynthesis [21]. In contrary to the reduction in chlorophyll content that was observed (Table 2), heightened regulation of the major chlorophyll enzymes and the enzymes involved in carbon fixation and carbon uptake were found, especially chlorophyll-ab-binding proteins (CBP), light harvesting proteins (LCBP), dehydroascorbate reductase (DHAR), Rubisco, and Rubisco activase (RuBA) (Additional file 5: Figure S5, Additional file 6: Figure S6, and Additional file 7: Table S1). A recent report by Valenzuela *et al.* [26] revealed that multiple carbon fixation pathways were activated during lipid accumulation in the marine diatom *P. tricornutum*. Valenzuela *et al.* [26] reported that when cell growth slowed and dissolved inorganic carbon (DIC) became more available during the nutrient-depleted conditions, carbon concentrating mechanism (CCM) may not be needed, but biophysical CCM could still increase CO_2_ flux to Rubisco by facilitating bicarbonate transport into chloroplast. This mechanism would potentially concentrate CO_2_ in the lumen surrounding the plastid for increased delivery to Rubisco. Some molecular components associated with the CCM have been identified [29,30], however, the association between the Rubisco function and CCM activity in *Chlamydomonas* had not previously been proven. Recently, Meyer *et al.* [31] reported that α-helices of Rubisco small subunit control pyrenoid formation in *Chlamydomonas* and the line between the pyrenoid and a CCM improves the operating efficiency of carbon assimilation and overcomes diffusive limitations in aquatic photosynthesis. In evolutionary terms, the activity of a CCM in *Chlamydomonas* involves intermediate specificity of Rubisco [31]. It has been suggested that the operational efficiency of CCM in *Chlamydomonas* can be determined or correlated with the evolution of Rubisco [31]. In addition, 6-phosphogluconate dehydrogenase (6PGDH), involved in the oxidative pentose-phosphate pathway (OPPP), was found to be overexpressed (Additional file 7: Table S1). 6PGDH reportedly oxidizes the glucose-6-phosphate into 6-phosphoglucolactone and further yields 6-phosphogluconate followed by Calvin cycle protein ribulose-5-phosphate (Ru5P) [4]. Interestingly, triose phosphate isomerase (TPI) was up-regulated, which is key enzyme that converts the triose phosphate (G3P) of Calvin cycle into dihydroxyacetone phosphate (DHAP). This reversible reaction (conversion of G3P into DHAP) is a key step for glycolysis and gluconeogenesis in chloroplasts [4]. These observations strongly support the hypothesis that detailed knowledge on carbon partitioning as nutrient conditions change is important to predict carbon flow for lipid synthesis [26]. When microalgal strains were allowed for adaptation under nutrient starvation conditions, multiple changes occurred in proteins involved in carbon, nitrogen, and energy metabolism. These proteins were involved in both cellular development and lipid accumulation metabolisms. For example, under continuous nitrogen starvation conditions, carbon metabolism in *C. reinhardtii* shifted from glucose synthesis to utilization and storage as starch [28]. It has been reported that four central enzymes were involved in microalgal carbon fixation, highly regulated under continuous light stress conditions [32]. These included phosphoribulokinase, phosphoglycerate kinase, glyceraldehyde-3-phosphate dehydrogenase, and triose phosphate isomerase [32]. It is notable that triose phosphate isomerase (TPI) was also significantly overexpressed in our study. The abundance of carbon fixation proteins during constant stress conditions increased lipid synthesis and storage. Furthermore, this lipid accumulation caused slower growth and reduction in photosynthetic pigments (note the reduction in chloroplast fluorescence under nitrogen starvation). However, the details of this process or linkage are still unknown and it has recently been suggested that a deeper understanding of carbon flux throughout the full range of cellular processes is necessary to optimize both growth and lipid accumulation in microalgal cultures [22]. In our study, the enzymes involved in nitrogen/carbon balance metabolism are up-regulated during adaptive evolution. The key control protein (periplasmic L-amino acid oxidase (LAO1)) of carbon-nitrogen integration was specifically overexpressed (Figure 6). This enzyme reportedly releases ammonium in addition to providing a supply of carbon backbones that feed back into the TCA cycle and acetyl-CoA [21]. A very recent study by Wase *et al*. [28] also found that the abundance of LAO1 highly increased in *C. reinhardtii* during nitrogen starvation conditions. It is suggested that the ammonia released by LAO1 could be transported into the plastid and assimilated to produce glutamate. Glutamate is one of the key molecules that connect the amino acid metabolic pathways to the carbohydrate and lipid biosynthetic pathways [28]. The majority of our proteomics data is in line with a recent iTRAQ based proteomics study of *C. reinhardtii* under nitrogen starvation conditions [28], which also finds a higher regulation of enzymes involved in carbon and nitrogen cycles, especially 6PGDH, TPI, and ALD. If we combine these results, it is conceivable that predicting the exact role of elements involved in an algal CO_2_-concentrating mechanism (CCM) can be used to enhance lipid productivity in algae. In addition, the major enzyme involved in glyoxylate shunt was also found to be overexpressed; the overexpression of glyoxylate shunt enzyme such as isocitrate lyase (ICL), and the overexpression of NADP dependent-malate dehydrogenase (NDH) enzyme which plays crucial role in chloroplast C4 cycle, supports the increase of the capacity to produce carbon backbone substrates [21]. Glutathione-S-transferases (GST) and esterase (EST), which are enzymes involved in lipid metabolism, were overexpressed during adaptive evolution. Most of the other identified proteins from our study were identical with those proteins identified from lipid-body proteomics [19,20]. These include Ran-like small GTPase (GTPase), ribosomal protein Sa (RPSA), peptidyl-prolyl cis-trans isomerase (PPlase), Rab GDP dissociation inhibitor protein (Rab GDP), aldehyde dehydrogenase (ALD), gamma-hydroxybutyrate dehydrogenase (GHB), and α and β-tubulin proteins (α-tub/β-tub; Additional file 7: Table S1). We are currently studying the changes and correlations between genetic (inheritable) alterations and the physiological acclimation of microalgal strains during adaptive evolution period. Overall, these molecular results from our adaptive evolution study demonstrate the potential role of photosynthesis performance in line with carbon partitioning, flux, fixation, and carbon/nitrogen metabolism during lipid accumulation in microalgae under nitrogen starvation.

## Conclusions

*C. reinhardtii* strains CC124 and sta6-1 have been subjected to adaptive evolution to understand the molecular mechanisms of lipid accumulation under nitrogen-depleted conditions. The algal population that exhibited an enhanced accumulation of lipids were specifically selected and enriched through several cycles of adaptive evolution using FACS. The adaptive evolution under nitrogen starvation through sorting and regeneration of high-lipid content cells led to the generation of lipid overproducing populations of *C. reinhardtii* CC124 and sta6-1. The results on lipid composition, elemental analysis, confocal microscopic analysis, and chlorophyll analysis reported here highlight the characterization of lipid accumulation with carbon/nitrogen metabolic pathways. Studies on proteomics further support the phenotypic characterization and reveal the potential role of carbon/nitrogen metabolic pathways in line with lipid accumulation. These results demonstrate that the adaptive evolution of microalgae can be a new tool that could be utilized to develop *C. reinhardtii* strains, or other microalgal strains with desired phenotypes, such as high lipid accumulation.

## Materials and methods

### Strains and culturing conditions

*C. reinhardtii* (wild-type CC124) was kindly provided by the Korea Research Institute of Bioscience and Biotechnology and *C. reinhardtii* mutant (cc-4348 sta6-1) was purchased from Chlamydomonas Resource Center, University of Minnesota, St. Paul, MN, USA. Both strains were grown to late-log phase in nitrogen-replete tris acetate phosphate liquid medium (TAP) [33] (approximately 2.0 × 10^6^ cells ml^−1^) and considered as 0 hour seeds. Zero hour seeds were re-suspended in a fresh TAP medium and taken immediately after re-suspension for FACS sorting (details available in flow cytometry and cell sorting section). Cultures were maintained at 27°C under a light intensity of 125 μmol/m^2^s with an agitation speed of 120 rpm. Samples for analysis were taken at the indicated times. Optical density was measured using a UV spectrophotometer (Beckman-Coulter DU 730 Life Science, California, United States) and cell counts were measured by hemocytometer (Neubauer-improved bright line, Marienfeld, Lauda-Konigshofen, Germany).

### BODIPY staining

BODIPY 505/515 (4,4-difluoro-1,3,5,7-tetramethyl-4-bora-3a,4a-diaza-s-indacen), was purchased from Sigma Aldrich (St. Louis, Missouri, USA). BODIPY 505/515 staining was performed as reported previously [13]. A 5 mM BODIPY 505/515 stock was prepared by dissolving in dimethyl sulfoxide (Sigma Aldrich, St. Louis, Missouri, United States) and stored in the dark. For efficient staining of the intracellular lipid bodies of *C. reinhardtii* strains, an aliquot of 0.2% DMSO (vol/vol) was added to the microalgal suspension. A total of 2 μL of BODIPY 505/515 stock solution was added into the l ml of algal-DMSO suspension and agitated for 1 minute on a vortex mixer (Scientific Industries Inc., Bohemia, New York, USA). Samples were then incubated in darkness for 5 minutes at room temperature. After the incubation period, the samples were directly used for FACS and microscopic studies.

### Flow cytometric analysis and cell sorting

A high speed flow cytometer, MoFlo XDP (Beckman Coulter, Fullerton, California, United States) was used for the analysis of cell staining and cell sorting. The fluorescence reading was obtained using an excitation of 488 nm with an argon laser (Beckman Coulter, Fullerton, California, United Sates). The emission signal was measured in channels upon excitation (FL1 channel centered at 530/40 nm and FL5 channel centered at 740 LP). The samples mean fluorescence intensity values and images were analyzed using SUMMIT Software Version 5.2 (Beckman Coulter, Fullerton, California, United Sates). The FACS settings of all channels were the same for all sorting procedures. Coulter Isoton II Diluent fluid (Beckman Coulter, Brea, California, United States) was used in all experiments as the flow cytometry sheath fluid. Cell sorting was carried out using cell sort precision mode, with a 70 μm nozzle. The 0 hour seed cell population was divided into three groups (in Figure 1: green, blue, and red color indicating low, average, and high lipid content cells, respectively) in the plot obtained based on two-dimensional dot plot (FSC, represented cell size) versus green fluorescence (FL1 represented neutral lipids; Figure 1). A total of 25,000 cells of the top 2% of the total population (from red group R4 regions) were sorted directly into the sterilized tubes containing 1 ml of nitrogen-supplemented TAP broth and re-suspended in nitrogen-replete or nitrogen-depleted TAP medium, separately. Samples were taken for cell count analysis at different intervals and cells were maintained until they reached high cell density (or late-stationary phase). A portion of high cell density (or late stationary phase) cells were screened using flow cytometry and 25,000 cells from the top 2% cells were sorted into the sterilized tubes containing 1 ml of TAP broth. Cultures were maintained at 27°C under a light intensity of 125 μmol/m^2^s with an agitation speed of 120 rpm. Triplicates were maintained for both wild-type CC124 and mutant sta6-1 strains, separately. The same protocol was followed for all indicated times.

### Total lipid and fatty acid analysis

The total lipids were extracted from the 10 mg of lyophilized biomass with a chloroform-methanol (2:1 v/v) solvent mixture (Merck, Darmstadt, Germany) using a procedure similar to the Folch’s method [34]. Fatty acid methyl esters (FAMEs) were produced from the extracted lipid by a transesterification reaction. Briefly, methanol was added to the extracted lipid with sulfuric acid as a catalyst and a transesterification reaction was allowed to occur at 100°C for 10 minutes. After the reaction, 1 ml of deionized water was added and the organic phase was separated from water phase by centrifugation at 4000 rpm for 10 minutes. A total of 1 ml of chloroform containing 0.5 mg of heptadecanoic acid (C17:0; Sigma Aldrich, St. Louis, MO, USA) was added to each tube as an internal standard. The FAMEs in organic phase were analyzed by gas chromatography (HP5890, Agilent, Santa Clara, CA, USA) with a flame ionized detector (FID) and INNOWAX capillary column (Agilent, Santa Clara, United States, 30 m × 0.32 mm × 0.5 μm).

### Microscopic determination of lipids in microalgal cells using BODIPY 505/515

A laser scanning confocal microscope (Eclipse C1si, Nikon, Kanagawa, Japan) with the excitation set at 488 nm and the emission set at 570 to 590 nm was used for selectively detecting microalgal lipid bodies stained with BODIPY 505/515 fluorescent dye. In order to calculate the number of lipid bodies in the cells, a liquid portion of cultures were stained by BODIPY 505/515 stain, fixed, and examined under confocal microscopy. Calculations of numbers of lipid bodies were made using micrographs of the intact cells and lipid bodies were scored.

### Elemental analysis

The lyophilized microalgal biomasses were used for elemental analysis. Carbon (C), hydrogen (H), nitrogen (N), and sulfur (S) contents were analyzed using FLASH 2000 series (Thermo Scientific, Waltham, Massachusetts, United States).

### Protein extraction and two-dimensional gel electrophoresis

*C. reinhardtii* CC124 and sta6-1 cells collected at indicated intervals were lyophilized and used for total protein preparation. Briefly, cells were harvested by centrifugation at 1130 g for 5 minutes at 4°C in pre-weighed tubes. The cell pellets were collected and washed twice with sterilized distilled water and frozen at −80°C, followed by vacuum freeze drying for 3 days. Lyophilized (150 mg of CC124 and 100 mg of sta6-1) samples were suspended in Hepes-KOH buffer (25 mM, pH 7.5) containing 1 mM PMSF (PhenylMethylSulfonyl Fluoride) and protease cocktail. The suspension was finely homogenized with a sterilized motor-pistol and centrifuged (13000 g for 30 minutes at 4°C). The supernatant was carefully collected and mixed with 100% acetone (Junsei Chemical Co. Ltd., Tokyo, Japan) (1:3 ratio) for protein precipitation. The mixture was incubated at −24°C for 12 hours. After incubation, the mixture was centrifuged (13000 g for 40 minutes at 4°C), and the protein pellet was collected and air dried until complete evaporation of acetone. The pellet was re-suspended in a minimum amount of rehydration buffer (8 M urea, 2 M thiourea, 4% CHAPS, 5 mM magnesium acetate, DTT and protease inhibitor cocktail). The re-suspended material was centrifuged at 13000 g for 15 minutes at 18°C and the supernatant was carefully collected, protein concentration was determined, and samples were stored at −80°C. The amount of protein was determined with a Bio-Rad Protein Assay Kit (Bio-Rad, Hercules, California, USA) using bovine serum albumin (BSA) (Thermo Scientific, Rockford, IL, USA) as a standard. For two-dimensional gel electrophoresis, 75 μg of protein samples were used. Isoelectric focusing (IEF) was performed using Ettan IPGphorII (Amersham Biosciences, San Francisco, California, United States). Equilibrated strips were inserted on SDS-PAGE for protein separation and silver staining was carried out.

### Image analysis

Quantitative analysis of digitized images was carried out using the PDQuest (Version 7.0, BioRad) software according to the protocols provided by the manufacturer. The quantity of each spot was normalized by total valid spot intensity. Protein spots were selected for the significant expression variation deviated over two fold in its expression level compared with control or normal sample.

### MALDI-TOF and protein identifications

All chemicals used in the protein identification study were of analytical grade (sodium bicarbonate, 4-Sulfophenyl isothiocyanate, α-cyano-4-hydroxycinnamic acid (CHCA), ammonium bicarbonate (Sigma Aldrich, St. Louis, Missouri, United States)). For protein identification by peptide mass fingerprinting (PMF), protein spots were excised, digested with trypsin (Promega, Madison, Wisconsin, United States), mixed with α-cyano-4-hydroxycinnamic acid in 50% acetonitrile/0.1% TFA, and subjected to MALDI-TOF analysis (Microflex LRF 20, Bruker Daltonics, Billerica, USA) as described by Fernandez *et al.* [35]. Spectra were collected from 300 shots per spectrum over m/z range 600 to 3000 and calibrated by two point internal calibration using Trypsin auto-digestion peaks (m/z 842.5099, 2211.1046). Peak list was generated using Flex Analysis 3.0 (Bruker Daltonics, Billerica, USA). The threshold used for peak-picking was as follows: 500 for minimum resolution of monoisotopic mass, 5 for S/N. The search program MASCOT, developed by The Matrixscience (Matrix Science Ltd, London, UK) was used for protein identification by peptide mass fingerprinting. The following parameters were used for the database search: trypsin as the cleaving enzyme, a maximum of one missed cleavage, iodoacetamide (Cys) as a complete modification, oxidation (Met) as a partial modification, monoisotopic masses, and a mass tolerance of ± 0.1 Da. PMF acceptance criteria is probability scoring.

### Statistical analysis

Triplicates of samples were analyzed throughout the experiments. Statistical analyses were performed using SigmaPlot 10.0 software (Systat Software Inc., Chicago, IL, USA). The results were expressed as means of triplicate experiments. The results were expressed as means ± SD (standard deviation). Differences were considered significant at *P* <0.05.

## Additional files

Additional file 1: Figure S1. Two-dimensional gels stained with silver staining (pH 3 to 10) for CC124. Additional file 2: Figure S2. Two-dimensional gels stained with silver staining (pH 3 to 10) for sta6-1. Additional file 3: Figure S3. Two-dimensional gels stained with silver staining (pH 4 to 7) for CC124. Additional file 4: Figure S4. Two-dimensional gels stained with silver staining (pH 4 to 7) for sta6-1. Additional file 5: Figure S5. Proteome profiles of CC124 during adaptive evolution period. Additional file 6: Figure S6. Proteome profiles of sta6-1 during adaptive evolution period. Additional file 7: Table S1. Complete list of differentially expressed proteins of adaptive evolved *Chlamydomonas reinhardtii* cells.

## Abbreviations

6PGDH: 6-phosphogluconate dehydrogenase
ALDH: Aldehyde dehydrogenase
AtpD: ATP synthase
CBP: Chlorophyll-ab-binding proteins
CF1: beta subunit
DHAR: Dehydroascorbate reductase
Est: Esterase
GHB: Gamma-hydroxybutyrate dehydrogenase
GST: Glutathione-S-transferases
GTPase: Ran-like small GTPase
HP: Hypothetical protein
HSP70C: Heat shock protein 70C
ICL: Isocitrate lyase
LAO1: Periplasmic L-amino acid oxidase catalytic subunit
LCBP: Light harvesting proteins
mtCA: Mitochondrial carbonic anhydrase β type
mtTF-Tu: Mitochondrial translation factor Tu
NDH: NADP dependent-malate dehydrogenase
P5CR: Pyrroline-5-carboxylate reductase
PFL: Pyruvate-formate lyase
PP1: Protein phosphatase 1
PPlase: Peptidyl-prolyl cis-trans isomerase, FKBP-type
Pre Prt: Predicted protein
PRK: Phosphoribulokinase Rubisco activase
RabGDP: Rab GDP dissociation inhibitor protein
RPLP0: Acidic ribosomal protein P0
RPSA: Ribosomal protein Sa, component of cytosolic 80S ribosome and 40S small subunit
RuBA: Rubisco activase
RubisCo: Ribulose-1,5-biphosphate carboxylase/oxygenase large subunit
TLP: Thylakoid lumen protein
TPI: Triose phosphate isomerase
α-tub: α -tubulin 1
β-tub: β-tubulin
