# Supplementary material for: Systematically programmed adaptive evolution reveals potential role of carbon and nitrogen pathways during lipid accumulation in Chlamydomonas reinhardtii
Source: Biotechnol Biofuels. 2014 Sep 6;7:117. doi: 10.1186/s13068-014-0117-7 (PMC4174265; doi:10.1186/s13068-014-0117-7)
Supplement: Additional file 3: Figure S3. — Two-dimensional gels stained with silver staining (pH 4 to 7) for CC124. [file 13068_2014_117_MOESM3_ESM.pdf]

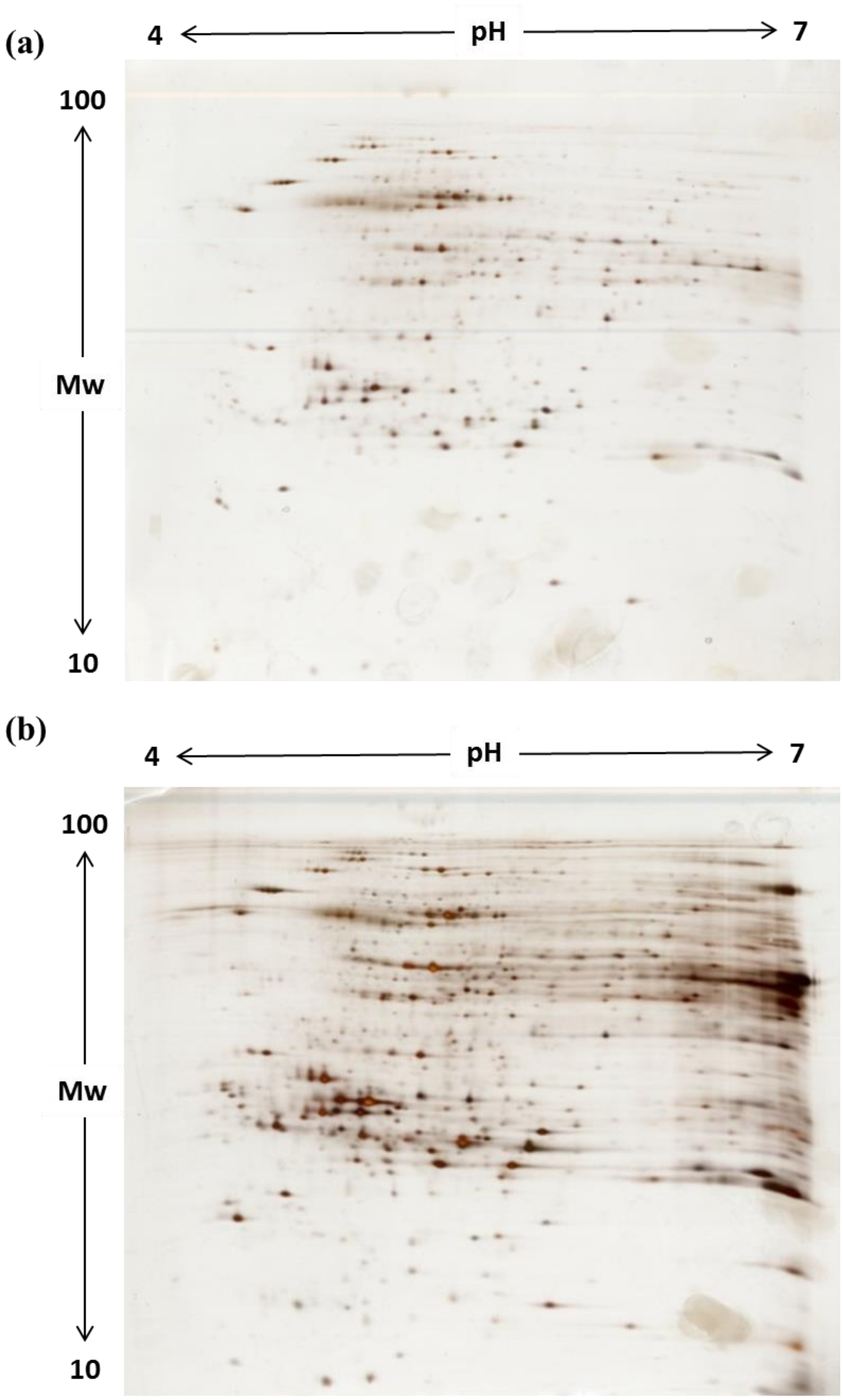

Figure S3. Two-dimensional gels stained with silver staining (pH 4-7) for CC124. Gels showing protein expression profiles of CC124 between different timing points (a) day 19 and (b) day 32, (c) day 62, and (d) day 77 during adaptive evolution.

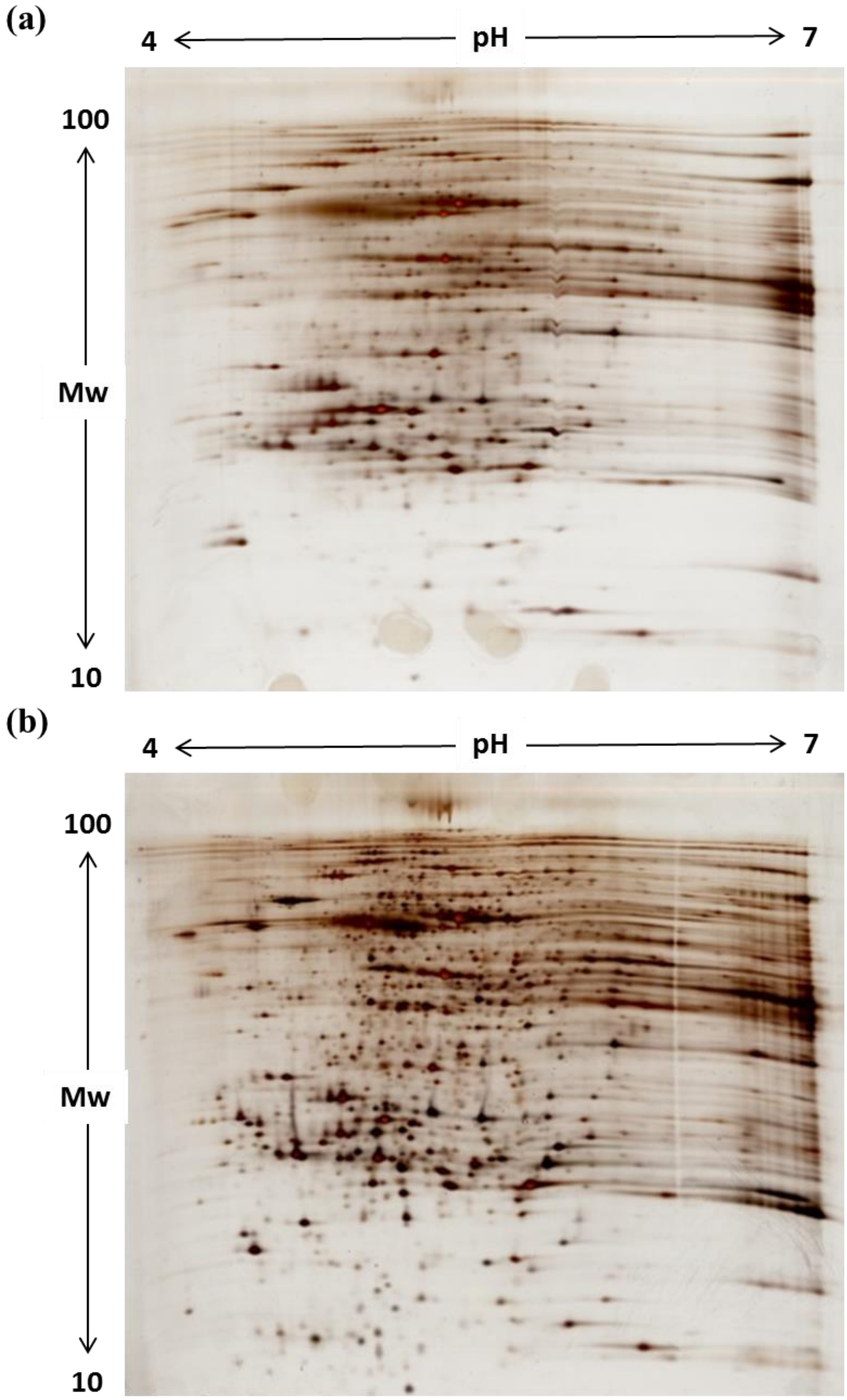

Figure S3. *(continued)* Two-dimensional gels stained with silver staining (pH 4-7) for CC124. Gels showing protein expression profiles of CC124 between different timing points (a) day 19 and (b) day 32, (c) day 62, and (d) day 77 during adaptive evolution.
