# Supplementary material for: Systematically programmed adaptive evolution reveals potential role of carbon and nitrogen pathways during lipid accumulation in Chlamydomonas reinhardtii
Source: Biotechnol Biofuels. 2014 Sep 6;7:117. doi: 10.1186/s13068-014-0117-7 (PMC4174265; doi:10.1186/s13068-014-0117-7)
Supplement: Additional file 4: Figure S4. — Two-dimensional gels stained with silver staining (pH 4 to 7) for sta6-1. [file 13068_2014_117_MOESM4_ESM.pdf]

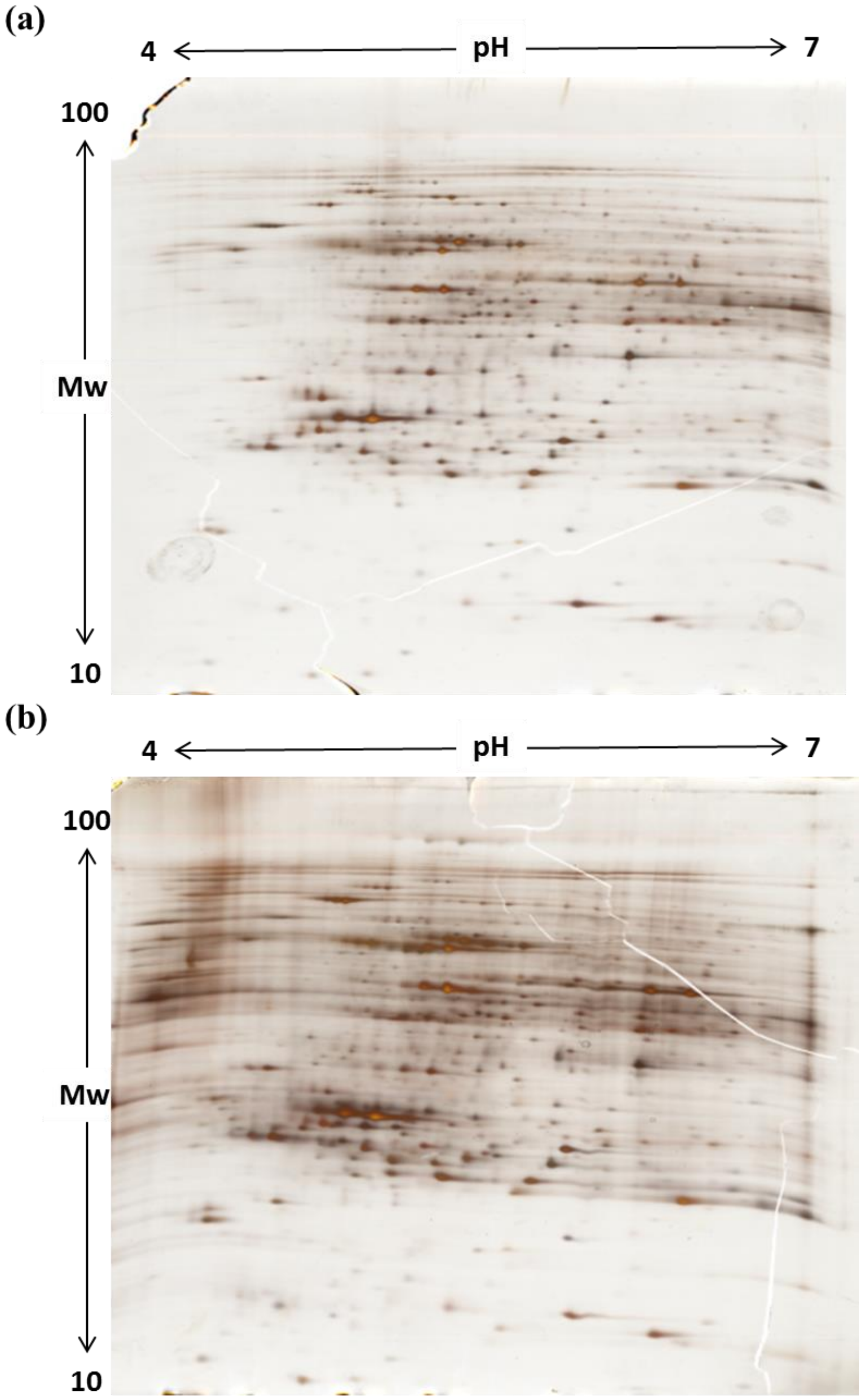

Figure S4. Two-dimensional gels stained with silver staining (pH 4-7) for sta6-1. Gels showing protein expression profiles of sta6-1 between different timing points (a) day 14, (b) day 27, and (c) day 43 during adaptive evolution.

(c)

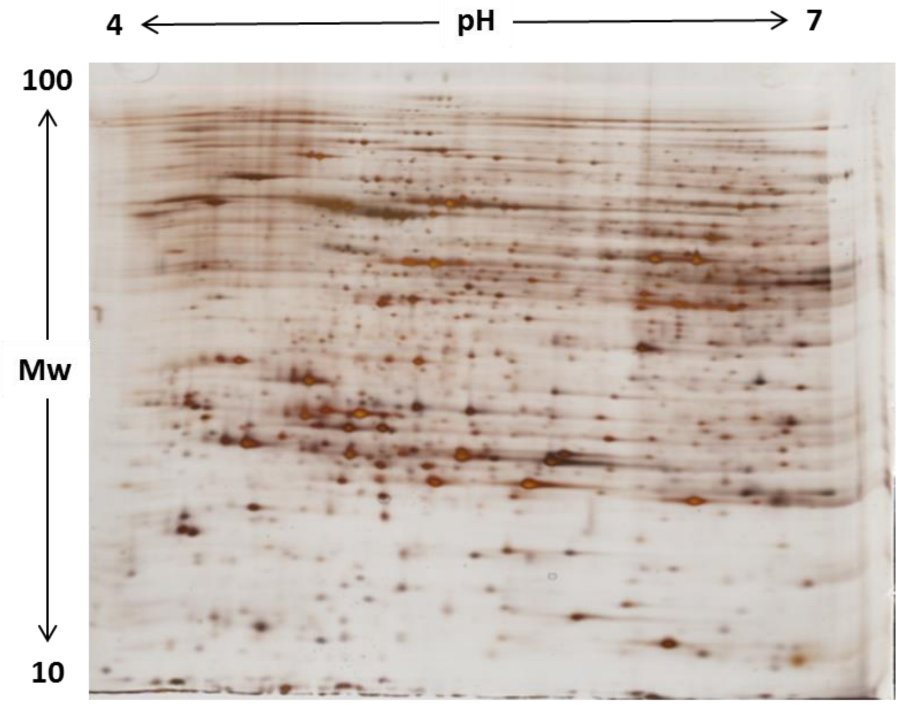

Figure S4. *(continued)* Two-dimensional gels stained with silver staining (pH 4-7) for sta6-1. Gels showing protein expression profiles of sta6-1 between different timing points (a) day 14, (b) day 27, and (c) day 43 during adaptive evolution.
