# Supplementary material for: Systematically programmed adaptive evolution reveals potential role of carbon and nitrogen pathways during lipid accumulation in Chlamydomonas reinhardtii
Source: Biotechnol Biofuels. 2014 Sep 6;7:117. doi: 10.1186/s13068-014-0117-7 (PMC4174265; doi:10.1186/s13068-014-0117-7)
Supplement: Additional file 5: Figure S5. — Proteome profiles of CC124 during adaptive evolution period. [file 13068_2014_117_MOESM5_ESM.pdf]

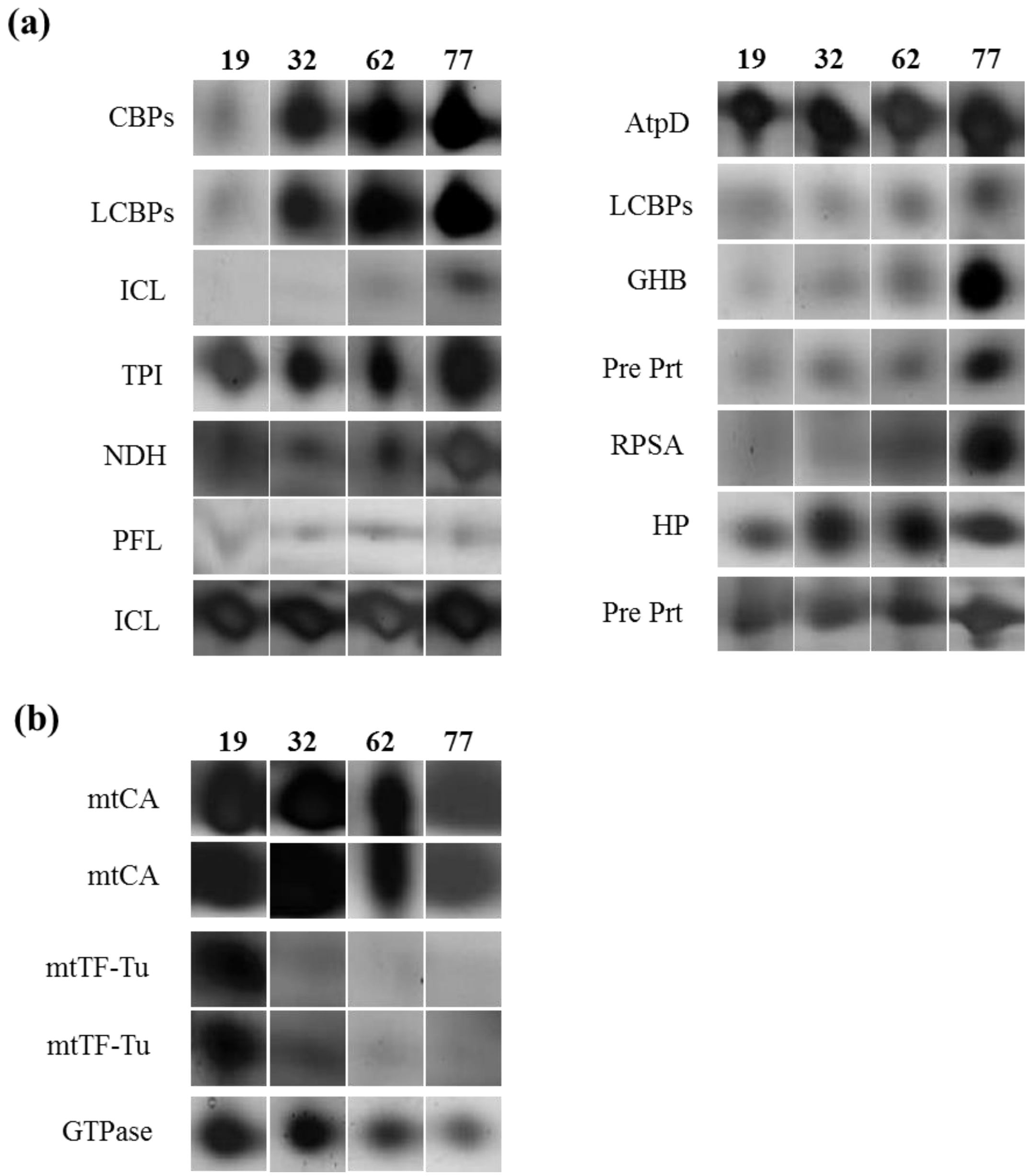

Figure S5. Proteome profiles of CC124 during adaptive evolution period. (a) The zoom-in image of protein spots which were (a) up-regulated or (b) down-regulated during adaptive evolution period. The abbreviations for the enzymes included are as follows: CBP, Chlorophyll-ab-binding protein of LHCII type I, chloroplast precursor; LCBP, Light-harvesting complex II chlorophyll a-b binding protein M3; ICL, Isocitrate lyase; TPI, Triose phosphate isomerase; NDH, NADP-Malate dehydrogenase: PFL, Pyruvate-formate lyase; AtpD, ATP synthase CF1 beta subunit; GHB, Gamma-hydroxybutyrate dehydrogenase; Pre Prt, Predicted protein; RPSA, Ribosomal protein Sa, component of cytosolic 80S ribosome and 40S small subunit: HP, Hypothetical protein; mtCA, Mitochondrial carbonic anhydrase β type; mtTF-Tu, Mitochondrial translation factor Tu; GTPase, Ran-like small GTPase.
