# Supplementary material for: Systematically programmed adaptive evolution reveals potential role of carbon and nitrogen pathways during lipid accumulation in Chlamydomonas reinhardtii
Source: Biotechnol Biofuels. 2014 Sep 6;7:117. doi: 10.1186/s13068-014-0117-7 (PMC4174265; doi:10.1186/s13068-014-0117-7)
Supplement: Additional file 6: Figure S6. — Proteome profiles of sta6-1 during adaptive evolution period. [file 13068_2014_117_MOESM6_ESM.pdf]

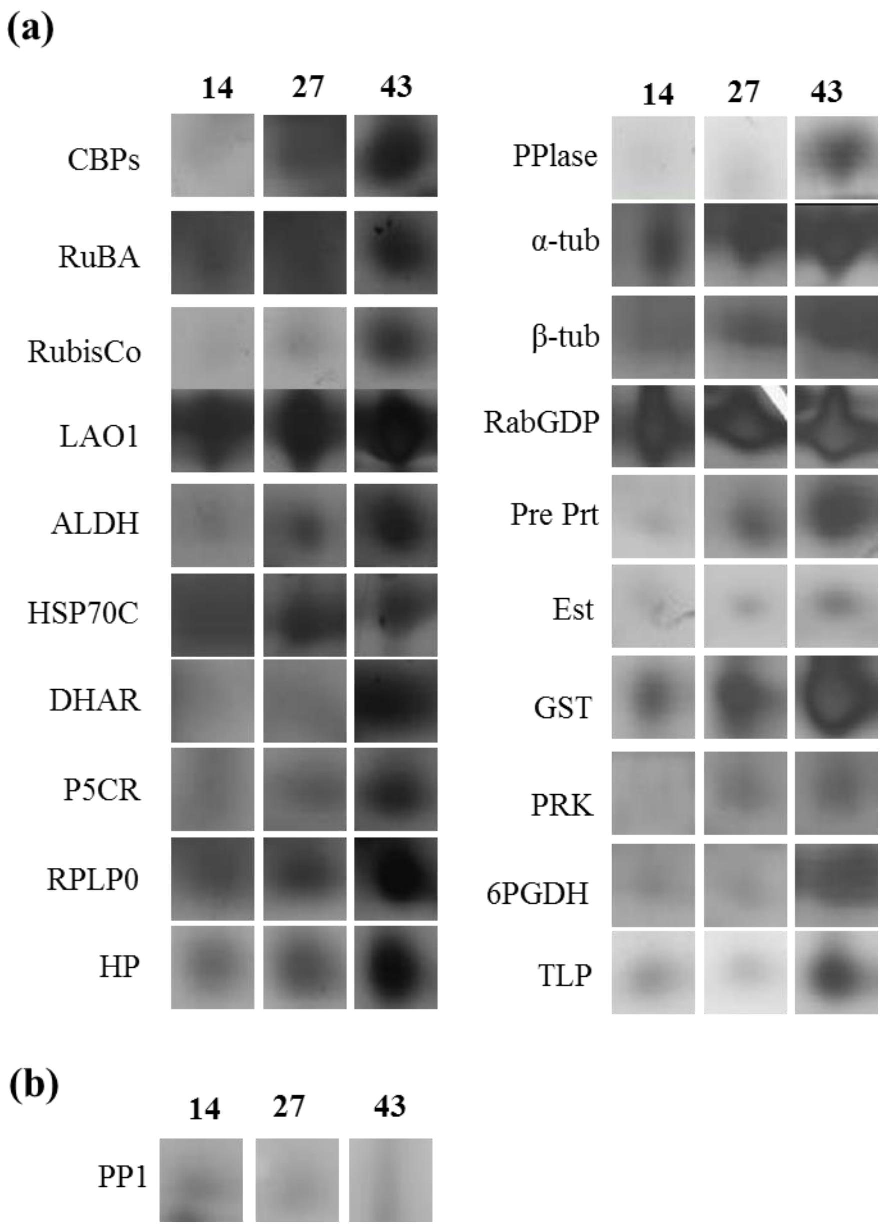

Figure S6. Proteome profiles of sta6-1 during adaptive evolution period. (a) The zoom-in image of protein spots which were (a) up-regulated or (b) down-regulated during adaptive evolution period. The abbreviations for the enzymes included are as follows: CBP, Chlorophyll-ab-binding protein of LHCII type I, chloroplast precursor; RuBA, Rubisco activase; RubisCo, Ribulose-1,5-biphosphate carboxylase/oxygenase large subunit; LAO1, Periplasmic L-amino acid oxidase catalytic subunit; ALDH, Aldehyde dehydrogenase; HSP70C, Heat shock protein 70C; DHAR, Dehydroascorbate reductase; P5CR, Pyrroline-5-carboxylate reductase; RPLP0, Acidic ribosomal protein P0; HP, Hypothetical protein; PPlase, Peptidyl-prolyl cis-trans isomerase, FKBP-type; α-tub, α -tubulin 1; β-tub, β-tubulin 2; RabGDP,; Pre Prt, Predicted protein; Est, Esterase; GST, Glutathione-S-transferase; PRK, Phosphoribulokinase Rubisco activase; 6PGDH, 6-phophogluconate dehydrogenase; TLP, Thylakoid lumen protein; PP1, Protein phosphatase 1.
