# Supplementary material for: Systematically programmed adaptive evolution reveals potential role of carbon and nitrogen pathways during lipid accumulation in Chlamydomonas reinhardtii
Source: Biotechnol Biofuels. 2014 Sep 6;7:117. doi: 10.1186/s13068-014-0117-7 (PMC4174265; doi:10.1186/s13068-014-0117-7)
Supplement: Additional file 7: Table S1. — Complete list of differentially expressed proteins of adaptive evolved Chlamydomonas reinhardtii cells. [file 13068_2014_117_MOESM7_ESM.docx]

**Table S1.** Complete list of differentially expressed proteins of adaptive evolved *C. reinhardtii* cells.

| No | Functional category and protein description | pI | Mr |
| --- | --- | --- | --- |
| 1 | Esterase (EST) | 5.90 | 31411 |
| 2 | NADP-Malate dehydrogenase (NDH) | 8.04 | 45280 |
| 3 | Pyruvate-formate lyase (PRL) | 6.49 | 91431 |
| 4 | Glutathione-S-transferase (GST) | 5.34 | 24000 |
| 5 | Glutathione-S-transferase (GST) | 5.34 | 24000 |
| 6 | Stress-related chlorophyll a/b binding protein 2 (CBP) | 4.88 | 28207 |
| 7 | Rubisco activase (RuBA) | 8.71 | 45229 |
| 8 | Rubisco activase (RuBA) | 8.71 | 45229 |
| 9 | Phosphoribulokinase Rubisco activase (PRK) | 8.11 | 42151 |
| 10 | Dehydroascorbate reductase (DHAR) | 6.34 | 25052 |
| 11 | Light-harvesting complex II chlorophyll a-b binding protein M3 (LCBP) | 5.68 | 27420 |
| 12 | Light-harvesting protein of photosystem I (LCBPs) | 9.12 | 26075 |
| 13 | Pyrroline-5-carboxylate reductase (P5CR) | 6.31 | 28608 |
| 14 | Acidic ribosomal protein P0 (RPLP0) | 6.07 | 34667 |
| 15 | ATP synthase CF1 beta subunit (AtpD) | 5.21 | 53243 |
| 16 | Isocitrate lyase (ICL) | 5.90 | 45948 |
| 17 | Isocitrate lyase (ICL) | 5.90 | 45948 |
| 18 | Mitochondrial translation factor Tu (mtTF-Tu) | 8.26 | 42942 |
| 19 | Mitochondrial translation factor Tu (mtTF-Tu) | 8.26 | 42942 |
| 20 | Rab GDP dissociation inhibitor protein (RabGDP) | 5.88 | 49720 |
| 21 | Peptidyl-prolyl cis-trans isomerase, FKBP-type (Pplase) | 6.06 | 11662 |
| 22 | Ribosomal protein Sa, component of cytosolic 80S ribosome and 40S small subunit (RPSA) | 5.07 | 30971 |
| 23 | Ran-like small GTPase (GTPase) | 6.24 | 25705 |
| 24 | Protein phosphatase 1 (PPI) | 5.08 | 35567 |
| 25 | 6-phophogluconate dehydrogenase (6PGDH) | 5.72 | 53617 |
| 26 | Predicted protein (Pre Prt) | 6.28 | 42690 |
| 27 | Thylakoid lumen protein (TLP) | 9.00 | 26710 |
| 28 | Hypothetical protein CHLREDRAFT_132186 (HP) | 6.30 | 29761 |
| 29 | Hypothetical protein CHLREDRAFT_184895 (HP) | 4.92 | 44286 |
| 30 | Ribulose-1,5-biphosphate carboxylase/oxygenase large subunit (Rubisco) | 6.29 | 45866 |
| 31 | Periplasmic L-amino acid oxidase catalytic subunit (LAO1) | 8.70 | 61272 |
| 32 | Periplasmic L-amino acid oxidase catalytic subunit (LAO1) | 8.70 | 61272 |
| 33 | Aldehyde dehydrogenase (ALD) | 7.30 | 59613 |
| 34 | β-tubulin 2 (β-tub) | 4.82 | 50157 |
| 35 | α-tubulin 1 (α-tub) | 5.01 | 50182 |
| 36 | Heat shock protein 70C (HSP) | 5.60 | 65435 |
| 37 | Chlorophyll-ab-binding protein of LHCII type I, chloroplast precursor (CBPs) | 5.96 | 27058 |
| 38 | Gamma-hydroxybutyrate dehydrogenase (GHB) | 5.22 | 29991 |
| 39 | Triose phosphate isomerase (TPI) | 7.56 | 30433 |
| 40 | Mitochondrial carbonic anhydrase β type (mtCA) | 9.04 | 28193 |
| 41 | Mitochondrial carbonic anhydrase β type (mtCA) | 9.04 | 28193 |
| 42 | Predicted protein (Pre Prt) | 4.90 | 33488 |
| 43 | Conserved hypothetical protein | 5.06 | 21304 |
| 44 | Predicted protein (Pre Prt) | 5.84 | 59842 |
